# Co-occurrence Patterns of Character Strengths and Measured Core Virtues in German-Speaking Adults

**DOI:** 10.3389/fpsyg.2020.599094

**Published:** 2020-11-26

**Authors:** Willibald Ruch, Sonja Heintz, Lisa Wagner

**Affiliations:** ^1^Section on Personality and Assessment, Department of Psychology at the University of Zurich, Zurich, Switzerland; ^2^School of Psychology, University of Plymouth, Plymouth, United Kingdom

**Keywords:** character strengths, virtues, VIA classification, VIA-IS, positive psychology

## Abstract

The VIA Classification on character strengths and virtues suggests 24 character strengths clustered into six core virtues (wisdom and knowledge, courage, humanity, justice, temperance, and transcendence). Three recent studies employed different methods for testing the assignment of character strengths to virtues (e.g., expert and layperson ratings), and generally supported the VIA classification. However, the co-occurrence of character strengths and virtues within individuals has not been examined yet. Another untested assumption is that an individual’s composition of character strengths is related to being considered of “good character.” Thus, the present study addresses three research questions: (1) How do character strengths and measured virtues co-occur within individuals? (2.1) How does the number of character strengths an individual possesses within a virtue cluster relate to their level of the respective virtue? (2.2) How does the composition of an individual’s character strengths relate to being considered of “good character”? We combined data from different studies to obtain a sample of *N* = 1,241 participants (*n* = 897 self-raters, *n* = 344 informant-raters, 70.1% female) aged 18 to 92 years (*M* = 30.64). All participants completed assessments of character strengths and virtues. Regarding (1), we found a high convergence of the correlations between strengths and virtues and the VIA Classification: 22 out of 24 character strengths correlated with the assigned virtue (exceptions were hope, which correlated highest with courage, and humor, which correlated highest with humanity). Also, 15 character strengths showed the numerically highest correlation with their assigned virtue. Regarding (2.1), overall, we found a linear trend between the number of strengths within one cluster and the virtue level. Regarding (2.2), we found higher levels of reported “good character” in those who possessed either (a) at least one character strength in each virtue cluster or (b) all character strengths in at least one virtue compared to those who did not. The present results contribute to the discussion regarding the structure of character: individuals’ character strengths relate to differences in virtues, across different measures and data sources. Relationships were mostly as expected, and deviations were consistent with results obtained using other approaches.

## Introduction

The VIA classification of character strengths and virtues (Peterson and Seligman, 2004) has sparked considerable interest in research and practice and is considered one of the major achievements and a cornerstone in positive psychology. A broad package of innovations was presented; for example a model of character, the identification of six core virtues from virtue catalogs, the concepts of character strengths and signature strengths, the list of criteria that define character strengths, a list of 24 character strengths that fulfill these criteria, an assignment of strengths to core virtues, and methods for assessing the strengths in different age groups. Of these, the measurement instruments received the most empirical attention. Both the VIA-IS (Peterson et al., 2005; for adults) and VIA-Youth (Park and Peterson, 2006; for children and youth) were used in various studies. Some of these studies were guided by the theoretical ideas suggested by Peterson and Seligman (2004), such as the postulate that character strengths contribute to various fulfillments that comprise the good life for the self and others. First, life satisfaction served as a proxy for fulfillment, and it was found that-while all strengths are fulfilling–the correlations were highest for hope, zest, love, gratitude, and curiosity (Park et al., 2004). This finding was also confirmed when using informant ratings (Buschor et al., 2013). Using broader conceptualizations of well-being (Hausler et al., 2017; Wagner et al., 2020a), a wider range of character strengths has shown robust and substantial correlations. Other studies were more exploratory; for instance, they determined the optimal factor structure for the 24 strengths or a subset of them (e.g., McGrath, 2014; Ng et al., 2017), or studied how character strengths relate to the Big Five personality traits (e.g., McGrath et al., 2020). Likewise, the VIA-IS was used to identify signature strengths, and participants were instructed to display them more often and in a new way (e.g., Seligman et al., 2005).

Peterson and Seligman (2004, p. 31; see also Peterson and Park, 2009) did not see the classification as a finished product, and they expected it to change: “We anticipate that our classification of strengths will (…) evolve, by adding or deleting specific strengths of character, by combining those that prove redundant, by reformulating their organization under core virtues and by more systematically evaluating them vis-à-vis our (…) criteria.” However, no strengths were added (although Peterson and Park discussed potential candidates) or deleted so far. This has proven to be a considerate decision in the light of McGrath et al.’s (2020) results, who showed that the scales were not redundant. Overall, as highlighted by Ruch and Stahlmann (2019), only very little empirical attention has been devoted to testing the assumptions put forward in the VIA classification. Stahlmann and Ruch’s (2020) test of the criterion that character strengths are morally valued represents an example for scrutinizing the classification’s claims. However, as also argued by Stahlmann and Ruch (2020), more testing of the basic premises of the VIA classification is urgently needed to build a more solid foundation for its further development.

In the present study, we aim at providing empirical tests of two central postulates included in the VIA classification (Peterson and Seligman, 2004): (1) Character strengths can be seen as distinguishable routes to displaying six core virtues (wisdom and knowledge, courage, humanity, justice, temperance, and transcendence), and (2) an individual can be considered as displaying a core virtue if one or two character strengths in the respective virtue are present, and an individual can be considered as having a “good character” if all six virtues are displayed at a certain level.

### The VIA Classification of Strengths and Virtues

An early think tank in search of the roots of a positive life initiated a research agenda on positive psychology and positive social science. One element was the outcomes of a good life (i.e., subjective fulfillment, objective fulfillment, and civic/societal recognition), enabling factors (social, genetic, human, and personal capital), and personal characteristics. The latter gradually developed into the VIA classification of character strength and virtues (Peterson and Seligman, 2004). Character was defined through 24 character strengths (positive traits that needed to fulfill most of 10 criteria to be accepted as a strength of character) and six “core virtues” (recurrent themes from virtue catalogs from different sources; see Dahlsgaard et al., 2005). The strengths were tentatively assigned to these core virtues (termed the “high six”) of wisdom and knowledge, courage, humanity, justice, temperance, and transcendence.

Table 1 shows that these clusters of strengths are also considered to share a common function (see also Ruch et al., 2019). For example, the strengths assigned to the core virtue wisdom and knowledge-creativity, curiosity, judgment, love of learning, and perspective-are considered cognitive strengths that entail the acquisition and use of knowledge. Likewise, love, kindness, and social intelligence are interpersonal strengths that cluster together as strengths of humanity, and their function is tending and befriending others (see Table 1 for the complete set of strengths and virtues). Peterson and Seligman (2004) speculate that the core virtues serve evolutionary functions, that is, that they have shown value for survival (see also Mayerson, 2020).

**TABLE 1:** Mean prototypicality of character strengths regarding the “High Six” (averaged across previous studies: Ruch and Proyer, 2015; Ruch et al., 2019; Giuliani et al., 2020).

	**Wisdom**	**Courage**	**Humanity**	**Justice**	**Temperance**	**Transcendence**
***Strengths of Wisdom and knowledge (“cognitive strengths that entail the acquisition and use of knowledge”)***						
Creativity	**64.70**	52.24	39.84	31.75	23.70	41.15
Curiosity	**65.05**	55.41	39.41	29.69	28.13	39.97
Judgment	**72.72**	48.71	45.55	51.32	46.17	35.08
Love of learning	**77.52**	52.73	37.20	32.94	30.71	39.54
Perspective	**79.20**	49.81	61.50	53.70	48.29	45.81
***Strengths of Courage (“emotional strengths that involve the exercise of will to accomplish goals in the face of opposition, external or internal”)***						
Bravery	43.37	**80.45**	51.38	47.58	40.43	35.77
Perseverance	56.39	**63.06**	31.00	34.56	47.92	29.97
Honesty	55.34	**66.15**	60.89	*66.23*	45.88	36.76
Zest	45.46	**65.70**	49.82	36.45	31.17	40.85
***Strengths of Humanity (“interpersonal strengths that involve tending and befriending others”)***						
Love	40.71	45.27	**83.94**	51.68	41.11	47.70
Kindness	40.39	39.53	**89.49**	59.27	36.94	46.36
Social Intelligence	61.89	46.98	**79.61**	57.19	44.53	38.88
***Strengths of Justice (“civic strengths that underlie healthy community life”)***						
Teamwork	47.06	44.53	*70.66*	**61.26**	44.04	38.86
Fairness	52.72	45.79	63.96	**80.80**	52.32	41.85
Leadership	*68.03*	*63.13*	60.58	**60.70**	45.84	40.66
***Strengths of Temperance (“strengths that protect against excess”)***						
Forgiveness	53.83	49.36	*78.20*	*58.81*	**57.72**	46.72
Humility	39.18	26.83	56.51	46.02	**60.45**	38.10
Prudence	*60.29*	34.38	36.32	33.27	**56.12**	26.23
Self-regulation	49.20	43.15	33.02	31.61	**77.00**	30.14
***Strengths of Transcendence (“strengths that forge connections to the larger universe and provide meaning”)***						
Beauty	49.06	29.16	43.58	26.27	27.13	**60.84**
Gratitude	50.94	39.65	*71.06*	51.97	46.30	**56.35**
Hope	48.79	*59.55*	47.99	37.87	39.56	**51.70**
Humor	*44.46*	*45.46*	*64.56*	*33.74*	*29.86*	**27.61**
Spirituality	39.83	39.77	49.89	34.83	36.83	**78.17**

*Average of four samples; ratings were rescaled to 0 to 100 where needed (Ruch and Proyer, 2015, and Study 2 in Ruch et al., 2019 used prototypicality ratings from 1 to 6). Beauty = Appreciation of beauty and excellence. Highlighted in boldface = rating on the assigned virtue. Italics = numerically higher ratings than on the assigned virtue.*

### The Relationships Between Character Strengths and Core Virtues

For over one decade, the question on the assignment of strengths to core virtues was not tested empirically. Recently, three studies have used different approaches to test the structure of the VIA classification, with overall converging results. In the latest of these studies, the highest and lowest participants’ strengths were determined, and then they were asked to remember and write down situations when they enacted these strengths in an excellent way (vs. normal way). The degree of presence of the six core virtues in these descriptions was rated subsequently by themselves as well as by 113 judges (Ruch et al., 2019; Giuliani et al., 2020; Study 1) and the averaged ratings allowed to see which virtue is typically believed to result from the excellent enactment of a certain strength. A further study (Ruch et al., 2019; Study 2) asked participants to rate the extent to which the respective strength fulfilled each of the functions (e.g., for humanity: “interpersonal strengths that involve tending and befriending others”) associated with the six core virtues as suggested by Peterson and Seligman (2004). Ruch and Proyer (2015) asked laypeople and experts of different fields to rate the 24 character strengths for their prototypicality for each of the six core virtues. Despite the methodological variety of these studies, the seemingly most straightforward finding is that the classification overall holds, but a few changes might be appropriate (e.g., humor seems not to be assigned correctly).

#### Criteria for the Assignment of Strengths to Core Virtues

However, in none of these studies the virtues were actually measured. We hence do not know whether individual differences in strengths actually correlate with individual differences in a core virtue; for example, whether the level of measured appreciation of beauty and excellence (beauty) would rise and fall together with the measured level of transcendence (i.e., the virtue it is assigned to, next to hope, humor, gratitude, and spirituality). This is the first aim of the present article.

We test this first aim using three criteria (A, B, and C). First, taking Peterson and Seligman (2004) as a starting point, we can expect that ideally, the correlation between beauty and transcendence should be high and the correlation between beauty and the other five core virtues should be low (i.e., this correlation should be highest in the row; criterion A). Second-and again ideally-because there are five strengths assigned to the virtue of transcendence, beauty should be among those five strengths that correlate most highly with transcendence (i.e., be among the highest in a column), while the others are low or close to zero (criterion B). For this criterion, we need to consider that the core virtues each have a different number of strengths (humanity and justice: three; courage and temperance: four; wisdom and knowledge and transcendence: five). The third criterion is testing which strengths have at least a small relationship (i.e., *r* ≥ 0.10 or prototypicality rating of ≥ 50) to the virtue they have been assigned to (criterion C). This shows which strengths fit at least to some extent to their core virtue, and which ones lack any relationship and seem “misfits.” Thus, we will have three ways of looking at any correlation between a strength and a virtue: one that compares this coefficient with others found for this strength (i.e., is any other core virtue more highly related?), one that compares this coefficient with others found for this virtue (i.e., is any other strength more highly related?), and one that focuses on the absolute value of the coefficient (i.e., does the strength relate at all to the core virtue?).

As another starting point or reference, we can examine what the published studies (Ruch and Proyer, 2015; Ruch et al., 2019; Giuliani et al., 2020) yielded so far. Leaving aside the patterns found by the individual studies, Table 1 shows the aggregated results found after the ratings were rescaled to run between 0 and 100.

Table 1 shows that all strengths of wisdom and knowledge fulfilled criterion A, as they were primarily prototypical for this very virtue. Perspective, love of learning, and judgment also fulfilled criterion B, as of all 24 strengths, these three were most strongly aligned with wisdom and knowledge. Curiosity and creativity followed at ranks 5 and 6, respectively, and leadership was on rank 4. Courage, bravery, perseverance, and zest met criterion A, but honesty was numerically slightly more related to justice. Bravery, honesty, zest fulfilled criterion B, and perseverance was 5th just slightly behind leadership. Love, kindness, and social intelligence fulfilled both criteria, A and B. All strengths of temperance met criterion B, and humility and self-regulation also fulfilled criterion A. Forgiveness, on average, related more strongly to humanity than to temperance and prudence more strongly to wisdom and knowledge than to temperance. Finally, all transcendence strengths (except humor) indeed fulfilled criterion B, and beauty and spirituality also fulfilled criterion A. However, gratitude and humor were higher on humanity, and hope was higher on courage than on transcendence. All strengths fulfilled criterion C, with the exception of humor (prototypicality of less than 50).

Leaving humor aside, there were only three violations for criterion B (leadership intruded into the clusters defining wisdom and knowledge and courage, and honesty was a better marker of justice than leadership and teamwork were), and seven violations for criterion A. This might be, in part, because some core virtues generally received lower ratings; the average of the three highest ratings was high for the two virtues that fully fulfilled criterion A: humanity (81.78) and wisdom and knowledge (75.12). It was intermediate for the two core virtues with some problems with criterion A (courage: 65.93; justice: 63.75), and lowest for those virtues where two and three strengths had violations (temperance: 59.09; transcendence: 58.59). While some core virtues may be simply less present in the strengths, it is also plausible that humanity and wisdom are clearer concepts to rate than transcendence and temperance. Such differences might affect rating studies, but they will be less of a problem when measures of the virtues are utilized. Consequently, the present study’s results will not only be interpreted regarding Peterson and Seligman’s (2004) assignment of strengths, but also the pattern found for the average of the four prior rating studies.

To address the gap in the current knowledge–the lack of data on the co-occurrence of character strengths and core virtues–the present study will investigate *research question 1*: How do the 24 character strengths of the VIA classification relate to the six core virtues when measured in individuals?

### How Many and Which Strengths Are Needed to Be Considered Virtuous?

If character strengths are considered “distinguishable routes to displaying (…) virtues” (Peterson and Seligman, 2004, p. 13), one might ask: Is pursuing one of these “routes” sufficient for displaying a virtue? Does the pursuit of more than one route lead to a stronger expression of a core virtue than the pursuit of only one of the routes? In their handbook, Peterson and Seligman (2004) put forward some quite general hypotheses regarding these questions. They argue that every character strength in each cluster is similar with regards to a shared function, but that an individual does neither need to display all of the character strengths in one cluster in order to be considered as showing a certain virtue nor all 24 character strengths to be considered of “good character.” However, these claims have, to our knowledge, never been tested empirically (see Ruch et al., 2019).

The first assumption put forward by Peterson and Seligman (2004) is that in order for an individual to be considered as virtuous concerning one of the core virtues, the individual should display one or two strengths out of the cluster of three to five strengths assigned to the respective virtue: “We are comfortable saying that someone is of good character if he or she displays but 1 or 2 strengths within a virtue group” (Peterson and Seligman, 2004, p. 13). For the present study, we use this assumption to derive *research question 2.1*: Does the number of character strengths within one virtue cluster relate to the level of the respective core virtue?

The second, related, premise presented by Peterson and Seligman (2004) is that in order for an individual to be considered of overall “good character,” such individual should display all six core virtues to a certain extent: “We speculate that all these virtues must be present at above-threshold values for an individual to be deemed of good character” (Peterson and Seligman, 2004, p. 13). In other words, the idea put forward is that a “good character” requires a balance between different virtues, and as a consequence a certain level of different strengths assigned to the different clusters. For the present study, we rely on this notion to derive *research question 2.2*: Are individuals who display at least one character strength of each of the six core virtues considered of “more good character” than individuals who do not display at least one character strength of each of the clusters? Alternatively, one might argue that ‘experts’ in certain virtues could also be considered of “good character.” Thus, we will also test whether individuals who possess all character strengths in at least one of the six core virtues report higher levels of “good character” than those who do not.

Designing an empirical test of both of these assumptions poses several challenges. Given the dimensional nature of the constructs, at what point can we say that someone is ‘displaying’ a strength? Given the variation in the number of character strengths assigned to each of the six core virtues (between three and five), does this perhaps mean that one strength is sufficient for virtues with only three strengths assigned to them and two strengths are necessary for virtues with four or five strengths assigned to them? In the present study, we will present an analytical approach that enables studying these relationships, but it is obvious that the relationships are very complex.

### Measuring Core Virtues?

Peterson and Seligman (2004) argued that the core virtues themselves cannot be measured because of their abstract nature. Therefore, they did not offer a measure for the core virtues, and they refrained from adding up the strengths for a virtue composite. While measures exist for individual core virtues (i.e., wisdom and knowledge, justice), their meaning does not match the definitions given in Peterson and Seligman (2004), and they often tend to be multidimensional. Therefore, an alternative way needs to be found for this study.

Following the footpath of Peterson and Seligman (2004), we assume in the present article that the 24 character strengths represent distinguishable routes to the core virtues. Individuals higher in a particular strength will enact this strength more often as the enactment of strengths is assumed to be gratifying. They will eventually get more skilled and improve this strength. More and more enactments will be excellent, and the resulting situations have virtue quality. This is exactly what the study by Giuliani et al. (2020) demonstrated: there was more excellent use of a strength among those for whom this character strength was a signature strength (i.e., a strength that is highly typical of an individual), and their written-up situations were rated as showing more expressions of a particular core virtue than the situations created by individuals who scored low in this strength. In these studies (Ruch et al., 2019; Giuliani et al., 2020), the virtue ratings were applied to two excellent and two everyday enactments, that is, to a very limited segment of behaviors. Core virtues can presumably be shown (or not shown) in a wider variety of situations and thereby enter the person’s self-concept, but the person will also earn the reputation to possess this virtue. Thus, a measure of the core virtue (self and informant) and its correlation with the strength will reflect the postulated path to the virtue as described by Peterson and Seligman (2004). Accordingly, the present study is a step further from Ruch et al. (2019) and Giuliani et al. (2020) as we assess the degree to which the virtues are displayed in general, which represents a more stable and reliable assessment of a person’s inclination to a particular core virtue. To ensure the respective virtue is understood the same way as in Peterson and Seligman (2004), we will use descriptions of the core virtues from the handbook and use quantifiers (e.g., how strongly they feel committed to this virtue, how fulfilling it is to act in line with this virtue) to allow for quantitative differences in the inclination to the core virtue.

### Aims and Overview of the Present Study

The present study addresses two main research questions: (RQ1) Which co-occurrence pattern emerges between character strengths and measured core virtues? We expect that individuals high in a particular strength will show actions or make decisions that will be seen as virtuous (by others and oneself), and the nature of the virtue ideally will be the one that may be predicted from the VIA-classification, resulting in a correlation between the character strengths and the respective core virtues. (RQ2.1) How does the number of character strengths displayed within each virtue group contribute to the level of this core virtue? Having no strength will make the enactment of a virtue difficult, but is enacting one strength sufficient, or is there a satiation point? (RQ2.2) How does the composition of an individual’s character strengths relate to being considered of “good character”? Is displaying at least one character strength of each virtue indicative of “good character”? And does displaying all character strengths of at least one core virtue suffice as well to be considered of “good character”?

## Materials and Methods

### Participants

Table 2 shows the characteristics of the four samples, including exclusions, demographics, and measures used. Samples 1, 2, and 3 consisted of individuals providing ratings about themselves, and Sample 4 consisted of individuals providing ratings about a close other (informant raters). The total sample was comprised of 1,241 adults (*M* = 30.64, *SD* = 13.65, range 18–92 years) and more females (70%) than males (30%). Exclusion criteria were age under 18 years, completion of less than 80% of the questionnaires, having more than 80% of the same responses (e.g., always selected “1”), being not fluent in German, not responding seriously (which was directly assessed by a question in Samples 3 and 4), and, for the informant ratings (Sample 4), not knowing the target well.

**TABLE 2:** Overview of the sample characteristics of the four samples including measures.

**Samples**	***Exclusions***	***Gender (M/F)***	**Age *M* (*SD*)**	**Education**	**Nationality**	**Measures**
Sample 1 (*N* = 260)	91	45.4%/54.6%	31.72 (12.14)	23.5% vocational training 24.6% university-entrance diploma 47.3% university degree	79.2% Swiss 13.8% German 1.2% Austrian 5.8% Other	CSRF ICV-7
Sample 2 (*N* = 378)	45	18.8%/81.2%	26.93 (10.81)	4.2% vocational training 72.2% university-entrance diploma 22.5% university degree	71.7% Swiss 23.3% German 1.1% Austrian 4.0% Other	VIA-IS ICV-6 GVR
Sample 3 (*N* = 259)	2	20.5%/79.5%	29.92 (13.43)	13.5% vocational training 52.5% university-entrance diploma 30.9% university degree	59.5% Swiss 35.9% German 2.7% Austrian 1.5% Other	VIA-IS CVRF GCR
Sample 4 (*N* = 344)	8	37.5%/ 62.5%	35.56 (15.74)	25.3% vocational training 32.2% university-entrance diploma 37.2% university degree	67.4% Swiss 26.5% German 3.2% Austrian 2.0% Other	CSRF informant-rating CVRF informant-rating GCR informant-rating

*CSRF, Character Strengths Rating Form; VIA-IS, VIA Inventory of Strengths; ICV-6/ICV-7, Inventory of Core Virtues; GVR, General Virtuousness Rating; CVRF, Core Virtue Rating Form; GCR, Good-Character Rating.*

Participants in Sample 1 were recruited by graduate psychology students attending a seminar on test construction at the University of Zurich (Switzerland), for which the students could obtain partial course credit (no reward was provided to the participants). Participants in Sample 2 were recruited by a master’s student, and participants could receive partial course credit (for psychology students), individual feedback on their results, and participate in a voucher lottery. Participants in Sample 3 were recruited by a master’s student and the third author and were asked to recruit two close others for the informant ratings (resulting in Sample 4, which consists of informant raters). These informant raters were then asked to complete the questionnaires with respect to the person who had invited them. They could receive individual feedback on their results and partial course credit (for psychology students), while there was no compensation for participants in Sample 4.

### Measures

#### VIA Inventory of Strengths (VIA-IS; Peterson and Seligman, 2004; German Version by Ruch et al., 2010)

It is the standard instrument to assess the VIA classification’s 24 character strengths. Each strength is assessed with 10 items and answered on a five-point Likert-type scale from 1 (very much unlike me) to 5 (very much like me). An example is “It is important to me that I live in a world of beauty” (appreciation of beauty and excellence). The validity and reliability (internal consistency and test–retest stability) of the German VIA-IS (Ruch et al., 2010) have been supported. In Sample 2, Cronbach’s alphas ranged from 0.71 (kindness) to 0.91 (spirituality), and in Sample 3 from 0.74 (honesty) to 0.89 (spirituality).

#### Character Strengths Rating Form (CSRF; Ruch et al., 2014)

The CSRF assesses the 24 character strengths entailed in the VIA classification with 1 item each. Each item consists of the character strength label (and synonyms if available) and a short description of the character strengths, followed by a rating from 1 (not like me at all) to 9 (absolutely like me). One example item is curiosity (interest, novelty seeking, openness to experience): “Curious people take an interest in all ongoing experience in daily life for its own sake and they are very interested in and fascinated by various topics and subjects. They like to explore and discover the world, they are seldom bored, and it’s easy for them to keep themselves busy.” The CSRF items have been shown to converge with the corresponding VIA-IS scales (correlations ranging between 0.44 and 0.77; Ruch et al., 2014).

#### Inventory of Core Virtues (ICV; newly developed for this study)

Short descriptions of each core virtue were developed by the authors and a group of psychology graduate students based on Peterson and Seligman’s (2004) descriptions. Each virtue description was presented on a different page, along with seven ratings that indicate the extent to which participants find the core virtue important, are committed to it, and act according to it. Participants answered each rating on a 10-point scale from 1 (not at all) to 10 (absolutely). In Sample 1, Cronbach’s alphas of the ICV-7 ranged from 0.93 (wisdom) to 0.98 (transcendence). In Sample 2, the ICV-7 was revised as follows, resulting in the ICV-6: the number of ratings was shortened from 7 to 6 (given very high reliabilities in Sample 1), and a negatively worded item was added (“This virtue is irrelevant to me.”). Additionally, the core virtue descriptions were adapted and shortened. In Sample 1, Cronbach’s alphas of the ICV-6 scales ranged from 0.93 (wisdom) to 0.98 (transcendence). The ICV-6 and ICV-7 are shown in the Supplementary Materials.

#### Core Virtue Rating Form (CVRF; newly developed for this study)

The CVRF is a short version of the ICV-6, containing one rating for each of the six core virtues on a nine-point scale from 1 (not at all) to 9 (completely) regarding the degree to which the virtue description describes the way participants typically behave (act, think, and feel). The CVRF is shown in the Supplementary Materials.

#### General Virtuousness Rating (GVR; newly developed for this study)

The GVR measures the degree to which someone can be considered to be generally virtuous. A short description of general virtuousness was developed by the authors and a graduate student. The ratings were adapted from the ICV-6, and the rating scale was the same. The GVR is shown in the Supplementary Materials.

#### Good-Character Rating (GCR; newly developed for this study)

The GCR measures the degree to which someone can be considered to be of “good character.” A description of the good character was developed by the authors and a graduate student. The rating was made on a nine-point scale from 1 (clearly not a good character [i.e., very vicious]) 9 “absolutely excellent/outstanding in character [i.e., good character without exception]”). The GCR is shown in the Supplementary Materials.

### Procedure

Samples 1 and 2 were collected online using the Unipark platform, and Samples 3 and 4 using the SoSci Survey platform. Participants in all samples completed other measures that are not relevant to the present study because they were collected as parts of larger projects. Samples 3 and 4 overlap with the samples used in Wagner et al. (2020a; Study 2) and Wagner et al. (2020b). However, the respective studies addressed different research questions and the overlap only refers to the self- and informant-rated character strengths. All samples were collected in line with the local ethical guidelines of the Ethics Committee of the Faculty of Arts and Social Sciences at the University of Zurich. All participants provided online informed consent.

### Analyses

The analyses were conducted using IBM^®^ SPSS^®^ Statistics Version 25 as well as R (R Core Team, 2020), using the packages haven (Wickham and Miller, 2020), dplyr (Wickham et al., 2020), rstatix (Kassambara, 2020a), emmeans (Lenth, 2020), ggplot2 (Wickham, 2016), and ggpubr (Kassambara, 2020b). Gignac and Szodorai’s (2016) effect size guidelines for research on individual differences were followed for the interpretation of correlations in research question 1, with correlations |0.10|–|0.19| as small, |0.20|–|0.29| as medium, and ≥ |0.30| as large. For the other analyses, the classic effect size guidelines by Cohen (1992) were followed.

## Results

Means and standard deviations of all scales (for both the single studies and the overall sample) are given in Supplementary Table S1. We decided to analyze the samples jointly while including the relevant methodological differences (character strengths measure, i.e., VIA-IS vs. CSRF, and information source, i.e., self- vs. informant ratings) as covariates (next to gender and age). To determine whether ratings of character strengths and core virtues converge (RQ1), the partial correlations (partialing out the control variables) between the 24 character strengths and the six core virtues were computed. Table 3 shows the partial correlations, and Table 4 shows the summary of the results according to the three criteria (A, B, and C).

**TABLE 3:** Partial correlations (controlling for gender, age, character strength measure, and information source) between the character strengths and virtue ratings across the four samples.

**No**	**CS**	**Wisdom**	**Courage**	**Humanity**	**Justice**	**Temperance**	**Transcendence**	***Mdn***
1	Creativity	0.19	0.18	0.03	0.07	0.04	0.07	0.07
2	Curiosity	0.19	0.19	0.06	0.15	0.09	0.06	0.12
3	Judgment	**0.30**	0.09	0.06	0.17	0.17	0.00	0.13
4	Learning	**0.25**	0.15	0.01	0.11	0.13	0.05	0.12
5	Perspective	**0.35**	0.12	0.13	0.15	0.18	0.09	0.14
6	Bravery	0.13	**0.39**	0.06	0.10	0.10	0.06	0.10
7	Perseverance	0.14	0.18	0.07	0.11	**0.25**	0.03	0.13
8	Honesty	0.15	0.16	0.19	**0.27**	0.16	0.08	0.16
9	Zest	0.12	**0.23**	0.10	0.11	0.12	0.06	0.11
10	Love	0.08	0.07	**0.28**	0.16	0.04	0.11	0.09
11	Kindness	0.06	0.07	**0.36**	**0.24**	0.10	0.11	0.10
12	Social Int.	0.16	0.11	**0.26**	0.18	0.16	0.10	0.16
13	Teamwork	0.01	0.07	**0.21**	0.17	0.13	0.06	0.10
14	Fairness	0.12	0.13	**0.29**	**0.40**	0.17	0.10	0.15
15	Leadership	0.15	0.19	0.12	0.15	0.16	0.05	0.15
16	Forgiveness	0.08	0.07	**0.20**	0.18	0.19	0.15	0.17
17	Humility	0.04	−0.02	0.19	**0.24**	**0.23**	0.13	0.16
18	Prudence	0.16	−0.03	0.12	0.19	**0.28**	0.07	0.14
19	Self-regulation	0.14	**0.20**	0.03	0.11	**0.39**	0.08	0.12
20	Beauty	0.15	0.13	0.15	0.12	0.12	**0.24**	0.14
21	Gratitude	0.10	0.14	**0.26**	**0.22**	0.15	**0.25**	0.18
22	Hope	0.09	0.19	0.08	0.08	0.11	0.07	0.09
23	Humor	0.09	0.13	**0.20**	0.12	0.05	0.02	0.11
24	Spirituality	0.03	0.10	0.08	0.02	0.06	**0.60**	0.07

*N = 1,241. Correlations ≥ |0.20| marked in bold. Correlations > |0.09| are significant at *p* < 0.001. CS = Character strengths, Learning = Love of learning, Social Int. = Social intelligence, Beauty = appreciation of beauty and excellence, Mdn = median correlation across the five virtues that the character strength is not assigned to.*

**TABLE 4:** Summary of the results in Table 3 in terms of the three criteria (A, B, and C) for the assignment of character strengths to virtues across the four samples.

**No**	**CS**	**Wisdom**	**Courage**	**Humanity**	**Justice**	**Temperance**	**Transcendence**
1	Creativity	ABC					
2	Curiosity	ABC					
3	Judgment	ABC					
4	Learning	ABC					
5	Perspective	ABC					
6	Bravery		ABC				
7	Perseverance		C				
8	Honesty		C				
9	Zest		ABC				
10	Love			ABC			
11	Kindness			ABC			
12	Social Int.			ABC			
13	Teamwork				C		
14	Fairness				ABC		
15	Leadership				C		
16	Forgiveness					C	
17	Humility					C	
18	Prudence					ABC	
19	Self-regulation					ABC	
20	Beauty						ABC
21	Gratitude						BC
22	Hope						–
23	Humor						–
24	Spirituality						ABC

*Learning = Love of learning, Social Int. = social intelligence, Beauty = appreciation of beauty and excellence. A = fulfilled criterion A (i.e., correlation was numerically higher than with the other five virtues). B = fulfilled criterion B (i.e., correlation was among the x highest in the column with x = number of strengths assigned to a virtue in the VIA classification). C = fulfilled criterion C (i.e., correlation of at least *r* = 0.10, which was significant at *p* < 0.001 and represented a small effect).*

Tables 3, 4 show that, using the total sample across the four samples, for the virtue of *wisdom and knowledge* all five strengths (creativity, curiosity, judgment, love of learning, and perspective) fulfilled all conditions; that is, their highest correlation was with wisdom/knowledge (criterion A), they were among the top-correlated strengths for wisdom/knowledge (criterion B), and their correlation with wisdom and knowledge was at least 0.10 (criterion C).

Regarding the virtue of *courage*, bravery and zest fulfilled all three criteria, but perseverance (higher on temperance) and honesty (higher on justice) were 7th and 9th, respectively.

Regarding the virtue of *humanity*, kindness, love, and social intelligence fulfilled all three criteria. Regarding the virtue of *justice*, fairness fulfilled all criteria; teamwork was higher on humanity, and leadership was higher on courage and temperance. Regarding criterion B, teamwork and leadership were 9th and 12th, respectively.

Two strengths of the virtue of *temperance* (self-regulation and prudence) fulfilled all three criteria. Forgiveness and humility fulfilled criterion C, but not A (forgiveness was correlated higher with humanity, prudence correlated higher with justice) or B (humility and forgiveness were ranked 4th and 5th, just behind perseverance).

Finally, beauty, and spirituality of *transcendence* fulfilled all criteria, and gratitude fulfilled B and C, but it correlated slightly higher with humanity (failing criterion A). Hope (highest with courage) and humor (highest with humanity) did not fulfill any criteria, with hope being on rank 13 and humor the second last of all.

Table 3 shows a few more peculiarities. First, 32% of the correlation coefficients were below 0.10; hence there was no relation at all between some strengths and virtues. Twenty-three correlations (16%) were larger than 0.20, and six were higher than 0.30 (i.e., medium and large effects, respectively). This shows that one strength for every virtue was particularly well-related to the virtue, namely spirituality for transcendence (0.60), followed by fairness for justice (0.40), bravery for courage (0.39), self-regulation for temperance (0.39), kindness for humanity (0.36), and perspective for wisdom (0.35). It is worth noting that transcendence and temperance – which had the lowest prototypicality scores of all virtues in previous studies (Ruch and Proyer, 2015; Ruch et al., 2019; Giuliani et al., 2020, see Table 1) – displayed the highest correlations of the respective character strengths with the core virtues. This shows that the previous studies’ limitation that these virtues were seemingly less well-represented (potentially originating from raters not being familiar with the concept) was overcome in the present study.

Additionally, the pattern of correlations was particularly similar for the core virtues of humanity and justice: the rank-order correlation of the correlations for these two virtues was 0.68 (*p* < 0.001), suggesting that strengths that predicted justice tended to also predict humanity.

### Character Strengths Predicting the Core Virtues

To test how well the strengths can predict the core virtues as an extension of RQ1, six hierarchical regressions were run. The core virtues were predicted by adding the control variables in Step 1 (i.e., age, gender, character strengths measure, and information source), the strengths theoretically assigned to one virtue in Step 2, and then adding the remaining character strengths in a stepwise fashion. The character strengths assigned to the core virtues always predicted additional variance beyond the control variables: wisdom and knowledge [Δ*F*_(__5_,_1229__)_ = 47.02, *p* < 0.001, Δ*R*^2^ = 0.155, total *R*^2^ = 0.191], courage [Δ*F*_(__4_,_1230__)_ = 59.38, *p* < 0.001, Δ*R*^2^ = 0.160, total *R*^2^ = 0.180], humanity [Δ*F*_(__3_,_123__)_ = 72.99, *p* < 0.001, Δ*R*^2^ = 0.141, total *R*^2^ = 0.239], justice [Δ*F*_(__3_,_1231__)_ = 80.34, *p* < 0.001, Δ*R*^2^ = 0.155, total *R*^2^ = 0.233], temperance [Δ*F*_(__4_,_1230__)_ = 68.16, *p* < 0.001, Δ*R*^2^ = 0.180, total *R*^2^ = 0.192], transcendence [Δ*F*_(__5_,_1229__)_ = 150.64, *p* < 0.001, Δ*R*^2^ = 0.366, total *R*^2^ = 0.406]. The amount of predicted variance was medium-sized for all core virtues and large for transcendence.

Next, we considered the individual predictors to determine which strengths from those assigned to a core virtue contributed most to the prediction. Significant predictors of wisdom and knowledge were judgment (β = 0.14, *p* < 0.001), love of learning (β = 0.10, *p* = 0.002), and perspective (β = 0.24, *p* < 0.001). Significant predictors of courage were bravery (β = 0.35, *p* < 0.001) and zest (β = 0.08, *p* = 0.006). Significant predictors of humanity were love (β = 0.10, *p* = 0.001), kindness (β = 0.27, *p* < 0.001), and social intelligence (β = 0.10, *p* = 0.002). The only significant predictor of justice was fairness (β = 0.40, *p* < 0.001). Significant predictors of temperance were forgiveness (β = 0.08, *p* = 0.007), humility (β = 0.08, *p* = 0.011), prudence (β = 0.10, *p* = 0.001), and self-regulation (β = 0.32, *p* < 0.001). Significant predictors of transcendence were appreciation of beauty and excellence (β = 0.08, *p* = 0.001), gratitude (β = 0.11, *p* < 0.001), hope (β = −0.11, *p* < 0.001), and spirituality (β = 0.57, *p* < 0.001). This shows that some core virtues were predicted to a similar extent by several assigned strengths (wisdom and knowledge, humanity, and temperance), while for others a clear “central strength” was found that predicted most of the variance in the core virtue (bravery for courage, fairness for justice, and spirituality for transcendence).

Finally, all core virtues except for wisdom and knowledge were predicted by additional strengths that were not theoretically assigned to them. Additional significant predictors of courage were fairness (β = 0.06, *p* = 0.040), prudence (β = −0.09, *p* = 0.003), and self-regulation (β = 0.10, *p* = 0.003), with Δ*R*^2^ = 0.011. Additional significant predictors of humanity were love of learning (β = −0.06, *p* = 0.020), fairness (β = 0.15, *p* < 0.001), gratitude (β = 0.09, *p* = 0.007), hope (β = −0.10, *p* = 0.001), and humor (β = 0.06, *p* = 0.039), with Δ*R*^2^ = 0.034. Additional significant predictors of justice were honesty (β = 0.12, *p* < 0.001), kindness (β = 0.06, *p* = 0.048), and humility (β = 0.08, *p* = 0.005), with Δ*R*^2^ = 0.022. An additional significant predictor of temperance was perseverance (β = 0.08, *p* = 0.010), with Δ*R*^2^ = 0.004. An additional significant predictor of transcendence was forgiveness (β = 0.06, *p* < 0.016), with Δ*R*^2^ = 0.003. Thus, the contribution of additional strengths to the prediction of core virtues beyond the theoretically assigned strengths was negligible in terms of effect sizes, with the exception of humanity and justice (small effects). However, there was no additional single strength that predicted these core virtues well, but rather a set of additional strengths that each contributed small amounts of additional variance.

### Strengths Possession and Core Virtues

**FIGURE 1:**
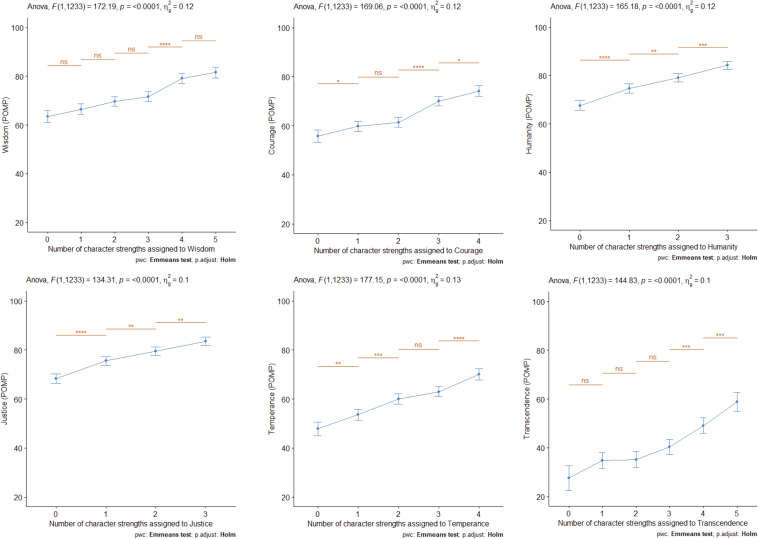
Ratings on core virtues in relation to the number of possessed character strengths in each virtue, POMP = percentage of maximum possible scores (range 0–100).

To test whether possessing additional character strengths of a core virtue contributes to higher scores in the core virtues (RQ2.1), we conducted six univariate ANCOVAs with the core virtues as dependent variables and the control variables (gender, age, character strength measure, and information source) as covariates. Predictors were the number of strengths assigned to the core virtue that the participants possessed. Strength possession was defined by a score above the grand mean in the respective strength. *Post hoc* comparisons of the different numbers of possessed strengths were conducted across adjacent strengths numbers, adjusted for multiple comparisons (Holm). Figure 1 shows the results and plots of the core virtue scores in relation to the number of displayed character strengths.

The number of strengths always significantly predicted the corresponding core virtues (all *p*s < 0.001) with medium-sized effects (10 to 13% explained variance). As can be seen in Figure 1, the more strengths participants possessed, the higher their corresponding core virtue scores were. The *post hoc* tests revealed that most notable increases in core virtue scores (significant for 5 of the 6 core virtues each) were found when comparing people who possessed all but one of the strengths with those who possessed all but two of the strengths of a core virtue, and those who possessed all strengths with those who possessed all but one of the strengths.

### Strengths Possession and the “Good Character”

Finally, to test the idea that “good character” requires either one strength from each core virtue or all strengths assigned to one core virtue (RQ2.2), we conducted two univariate ANCOVAs with “good character”/general virtuousness as dependent variables and the control variables (gender, age, character strength measure, and information source) as covariates. Predictors for the first ANCOVA were possessing vs. not possessing at least one strength of each virtue, and for the second ANCOVA possessing vs. not possessing all strengths of at least one core virtue. Strengths possession was again defined by a score above the grand mean in the respective strength. The assumptions were that participants who possess vs. do not possess at least one character strength of each core virtue, or those who possess vs. do not possess all character strengths of at least one core virtue, would score higher in the “good character” ratings. The descriptive statistics supported this notion for both the first assumption (*M* = 71.09, *SD* = 14.64, *n* = 520, vs. *M* = 63.85, *SD* = 14.88, *n* = 458) and second assumption (*M* = 70.94, *SD* = 14.80, *n* = 560, vs. *M* = 63.35, *SD* = 14.61, *n* = 418). The ANCOVA revealed significant, albeit small to medium differences, *F*_(__1_,_973__)_ = 57.97, *p* < 0.001, $\eta_{\text{p}}^{2}$ = 0.056, and *F*_(__1_,_973__)_ = 53.52, *p* < 0.001, $\eta_{\text{p}}^{2}$ = 0.052, respectively. Thus, both of the character strengths compositions that can be assumed to facilitate a “good character” were empirically supported with small to medium effects.

## Discussion

The present study aimed at investigating basic postulates regarding the VIA classification of strengths and virtues. The first one (RQ1) relates to the assignment of strengths to core virtues. After having relied on rating studies before (Ruch and Proyer, 2015; Ruch et al., 2019; Giuliani et al., 2020), the present study was the first to correlate strengths with measured virtues based on predictions coming from Peterson and Seligman (2004) and the results obtained in the previous studies. Strengths help enact morally excellent behaviors; for example, if fairness is enabling justice, scores in fairness should correlate with self- and informant-rated justice levels. This correlation should be the highest in the row (criterion A), but it should also be in the bulk of the highest coefficients in the column (criterion B), and at least of small magnitude (criterion C).

Fifteen strengths fulfilled all three criteria (creativity, curiosity, judgment, love of learning, perspective, bravery, zest, kindness, love, social intelligence, fairness, self-regulation, prudence, beauty, and spirituality); that is, their correlation with the virtue was the highest in the row and among the highest in the column and at least of small magnitude. These strengths span all six core virtues and can be seen as solid support for the classification. A few more (humility, forgiveness, and gratitude) only had minor deviations (e.g., one rank lower than another strength unaffiliated with this core virtue, which had a higher correlation with the virtue, or failing to be the highest correlation by a difference of 0.01). They can also be seen as supporting the classification, and we can conclude that, overall, 18 strengths did fit well.

How about the others? Humor and hope did not satify any of the three criteria. Humor should definitely be moved to humanity, unless the items are changed to capture transcendence. Humor can indeed be seen as an interpersonal strength that involves “tending and befriending others.” A series of studies has shown that humor is multidimensional, and certain contents might relate to any of the six core virtues, but humanity (and wisdom/knowledge) were the most frequent (Beermann and Ruch, 2009a,b). Hope could be seen as a candidate for courage, and its definition (“expecting the best and working to achieve it”) at least partially fits the description of an emotional strength that involves the “exercise of will to accomplish goals in the face of opposition, external or internal”. Taking the results of the rating studies as a starting point for this study was also justified; as in the prior studies, leadership was also marking wisdom/knowledge and courage, and honesty also related to justice. This was consistent across both approaches (see Table 1).

The initial classification was already quite valid, but some assignments need revision as foreseen by Peterson and Seligman (2004). While some changes seem well-justified, no final word can be spoken now, as more studies are needed. However, it seems obvious that the assignment of strengths to virtues can and must be empirically examined, that different methodologies may yield comparable results, and that a revision should also consider allowing the assignment of a strength to multiple core virtues.

The squared multiple correlations between strengths and virtues were between 14% and 37%, showing that strengths and virtues were overlapping, but different. The strengths together did not explain all reliable variance in virtues; thus, it does not seem right to simply add the strengths and treat them as a measure of core virtues. It may serve as a crude proxy, but a separate measurement of the virtues is preferable and feasible based on the present results.

The intercorrelation among the virtues followed a certain pattern: there was a higher correlation between humanity and justice (see Supplementary Table S2), as predicted by Peterson and Seligman (2004) and as found in prior studies (Ruch and Proyer, 2015; Giuliani et al., 2020). The other correlations were low but typically positive. This suggests that people committed to one core virtue tended to be committed to the others as well, but the virtues functioned well-independently from each other. Given the positive intercorrelation of the virtues, it is noteworthy that the pattern of correlations between strengths and virtues contained a lot of near-zero correlations, suggesting that there is indeed a pattern rather than a base rate of overlap due to unspecific effects.

It should be noted that the emerging consistency across the previous findings and the present study (i.e., Table 1) only draws from the correlation pattern of which strengths facilitate which virtue. Studies might consider testing whether training the strengths also increases the likelihood of the respective virtue to emerge. Further studies in a different context will build on the generalizability of these findings. However, it should be noted that the prime focus is here on the relationship between strengths and core virtues (or other desired outcomes). A different line of research focuses on the intercorrelations among the strengths to find a lower-dimensional space to still represent much of the reliable variance in the original strengths; that is, to find the essence in clusters of strength through the application of factor analysis. Such a research endeavor will likely discard strengths that do not show simple structure and move on to derive measures for the factors found, as the explanatory power is considered to be there, rather than in the many partly redundant lower-order traits. Such an approach leads to a parsimonious model and often produces a short instrument allowing to measure individual differences in character with few items, and there will be useful applications for this. However, when considering the prediction of meaningful outcomes, lower-order traits or even individual items have frequently demonstrated superior criterion validity (e.g., Dudley et al., 2006; Revelle et al., 2020).

The second postulate tested in the present study related to the number of strengths needed to display a virtue (RQ 2.1). We followed Peterson and Seligman (2004) in as much as we varied the number of strengths someone has (dichotomized test scores), but deviated from these authors as we measured the core virtue as a continuum (not as a dichotomy; i.e., having or not having a virtue).

The six core virtues showed distinct patterns: for the virtues of humanity and justice, we observed a relatively steep incline when comparing those individuals who possess no strength in this virtue cluster and those who possess one of the relevant strengths, which might be interpreted as partially supporting Peterson and Seligman’s (2004) claim of one strength being potentially sufficient to display the respective virtue. However, the virtue scores also increased from one to two and from two to three virtues, contradicting the idea of a satiation point. The patterns demonstrated by the core virtue of wisdom and knowledge and, to a certain extent, also by the virtue of courage, were consistent with the notion of a satiation point: in the pairwise comparisons, levels of wisdom/knowledge only increased significantly when comparing those individuals who possessed three strengths assigned to the respective virtue with those who possessed four strengths in the cluster. For courage, the increase in virtue scores was strongest when comparing the groups who possessed two vs. three strengths in the virtue cluster. Finally, temperance and transcendence showed yet a different pattern, with the strongest incline observed for the final steps from possessing three to four strengths (temperance) or from possessing three to four and four to five character strengths (transcendence). This pattern is more in line with the idea that one can achieve higher levels of a virtue if one possesses more of the character strengths assigned to one core virtue.

In conclusion, these results can be interpreted as offering some support for Peterson and Seligman’s (2004) claim that one character strength of the respective virtue cluster is sufficient for displaying the respective virtue: for four of the six core virtues, possessing one character strength was sufficient for a significantly higher virtue score when compared to possessing none of the relevant character strengths. However, there seems to be little support for the notion of a satiation point in general, as most virtue scores showed notable increases as more strengths were possessed.

We also tested whether possessing at least one character strength of each of the six virtue clusters or possessing all character strengths in one of the virtue clusters went along with higher scores in ratings of having a “good character” (RQ2.2). We found support for both the “balanced” assumption presented in Peterson and Seligman (2004) and the alternative, “expert” assumption. These results might be a starting point for further research considering the effects of the composition of character strengths and possible interactions between them.

Some limitations of this study warrant mentioning. First, the virtue measures were constructed *ad hoc* with a strong reference to the descriptions provided by Peterson and Seligman (2004), and depending on the context of the descriptions, some contents may be in the foreground. Second, participants came from only one cultural background, and future testing of the assumptions put forward in the VIA Classification should involve non-western countries as well. Third, in particular with regards to RQ2.1 and 2.2, the abstract claims made in the VIA classification made it impossible to test them directly. Due to the ambiguity and vagueness of their statements, our operationalization and analytical strategy might not fully reflect the ideas by Peterson and Seligman (2004). For example, when they refer to someone as being of “good character” when a certain number of strengths are present, they do not explicitly state that someone who possesses more strengths would be of better character if this were assessed dimensionally. Rather their statement might be interpreted to refer to the point when a threshold is being passed; that is, when displays of a strength turn virtuous. To test this, the assessment of virtue would need to be different and sensitive to differences in the threshold region.

## Conclusion

The present study helps to further the VIA model of character by empirically testing some of the most basic ideas put forward at its beginning (Peterson and Seligman, 2004). Research question 1 picked up the suggestion by the creators that the classification might change in the years to come. They mention specifically humor (“admittedly the most controversially placed entry”; p. 519) and foresee humanity as an alternative placement. Overall, both strategies, prototypicality ratings of concepts and empirical covariation of strengths and measured core virtues, seem to be viable ways to bring answers to this question. What is needed now is replication in other cultural contexts, and then the time will be ready to make more firm suggestions for a change in the classification. Research questions 2.1 and 2.2 opened questions relating to how many strengths are needed to enact a virtue and how core virtues related to a “good character.” Peterson and Seligman (2004) did not assume a simple linear model where strengths add up, but they considered configurations; that is, minimal numbers of strengths that are needed to enact a virtue. Likewise, they emphasized a balanced composition of core virtues. Answers to these questions are needed to understand what character is, but also for character development and training. We believe that it is important to review the work on the foundations of character, what character is and not only what it does. The contribution of this study to the field is that it highlights what was left to work on after Peterson and Seligman (2004) and to initiate some lines of research. This will eventually feed into developing character research further and also inform revisions of the strength and virtues classification and handbook.

## Data Availability Statement

The raw data supporting the conclusions of this article will be made available by the authors upon request, without undue reservation.

## Ethics Statement

Ethical review and approval was not required for the study on human participants in accordance with the local legislation and institutional requirements. The patients/participants provided their online informed consent to participate in this study.

## Author Contributions

WR initiated and conceptualized the studies. SH and LW supervised data collection. WR wrote the introduction and discussion, with contributions by LW. SH and LW analyzed the data. SH wrote the sections on methods and results with contributions from WR and LW. All authors helped in designing the studies and provided feedback and approved the final version of the manuscript.

## Conflict of Interest

WR is a Senior Scientist for the VIA Institute on Character, which holds the copyright to the VIA Inventory of Strengths. The remaining authors declare that the research was conducted in the absence of any commercial or financial relationships that could be construed as a potential conflict of interest.
