# Supplementary material for: Co-occurrence Patterns of Character Strengths and Measured Core Virtues in German-Speaking Adults
Source: Front Psychol. 2020 Nov 26;11:599094. doi: 10.3389/fpsyg.2020.599094 (PMC7726161; doi:10.3389/fpsyg.2020.599094)
Supplement: Supplementary file 1 [file Table_1.docx]

**Assessment of core virtues and the good character**

**Sample 1: German (original)**

**Inventory of Core Virtues (ICV-7)**

Der vorliegende Fragebogen befasst sich mit dem Thema Tugend. Unter Tugend versteht man eine hervorragende Eigenschaft oder vorbildliche Haltung. Im weitesten Sinn kann jede Fähigkeit, als wertvoll betrachtete Leistungen zu vollbringen, als Tugend bezeichnet werden. In der Ethik bezeichnet der Begriff eine als wichtig und erstrebenswert geltende Charaktereigenschaft, die eine Person befähigt, das sittlich Gute zu verwirklichen. Im Folgenden werden Ihnen sechs Tugenden vorgestellt und Ihnen verschiedene Fragen zu diesen gestellt. Bitte beachten Sie, dass es keine richtigen oder falschen Antworten gibt uns ist Ihre persönliche Meinung wichtig.

**Virtue descriptions**

Weisheit: Weisheit umfasst eine ausgeprägte Wissens-Breite und Tiefe über die Lebensbedingungen, menschlichen Angelegenheiten und Urteilsvermögen in der Anwendung/Umsetzung dieses Wissens. Es ist nicht die Intelligenz im Sinne eines IQ, sondern vielmehr eine praktische Intelligenz, die zu ausgewogenen guten Lösungen zwischen Eigen-, Drittinteressen und Kontext führt.

Tapferkeit: Tapferkeit kann auf verschiedene Arten gezeigt werden. *Physische Tapferkeit* bedeutet Ängste vor physischer Verletzungen oder vor dem Tod zu überwinden, um das Leben anderer oder sich selbst zu retten. *Moralisch tapfer* ist eine Person, die ethische Integrität und Authentizität (Treue zu sich selbst) bewahrt, selbst wenn sie damit den Verlust von Freunden, der Arbeit, der Privatsphäre oder von Prestige riskiert. *Psychologische Tapferkeit* braucht es für die Konfrontation mit einer schwächenden Krankheit, destruktiven Gewohnheiten oder Situationen.

Gerechtigkeit: Die Tugend Gerechtigkeit bezieht sich auf das, was wir als fair empfinden. Dieses Empfinden unterscheidet sich je nach kulturellem Hintergrund und persönlichen Vorstellungen. Gerechtigkeit wird oft mit der Vorstellung verknüpft, dass jeder erhält was er verdient, dass jeder die gleichen Chancen hat und dass die Ansprüche an die Bedürftigkeit angepasst werden.

Transzendenz: Die wörtliche Bedeutung stammt aus dem Lateinischen *transcendere*, was überschreiten heisst. Es ist das, was über das menschliche Wissen und den Verstand hinaus geht. In einem breiten Sinne ist es die Verbindung zu etwas höherem. Es ist der Glaube, dass es einen Sinn oder Zweck gibt, der grösser als der Mensch selbst ist. Im klassischen Sinne schliesst Transzendenz Begriffe wie Religiosität und Spiritualität mit ein. Transzendenz kann aber auch durch etwas oder jemanden ausgelöst werden, das/der die menschlichen Bedenken und das Selbst unbedeutend erscheinen lässt, und zu Ehrfurcht, Hoffnung sowie Dankbarkeit inspiriert.

Mässigung: Die Tugend Mässigung zu besitzen heisst, dass man Exzesse kontrollieren kann. Damit ist nicht nur die Kontrolle über Verlangen wie Essen, Trinken, Rauchen und Sex gemeint, sondern Mässigung beinhaltet jegliche Form von erfolgreicher Selbstbeherrschung. Mässigung umfasst somit die übergreifende Fähigkeit, seine eigenen Gefühle, Absichten und Verhaltensweisen kontrollieren zu können.

Menschlichkeit: Menschlichkeit bezeichnet eine zwischenmenschliche Stärke, die sich in der Fürsorge und in den Hilfestellungen für Mitmenschen zeigt. Die Menschlichkeit äussert sich in der Freundlichkeit, der Grosszügigkeit, dem Wohlwollen und dem Mitgefühl für andere. Personen, die eine hohe Ausprägung dieser Tugend aufweisen, sind sich den Gefühlen und Motiven von anderen und sich selbst bewusst und wissen, was in verschiedenen sozialen Situationen zu tun ist. Menschlichkeit äussert sich ebenfalls im Pflegen von engen Beziehungen und dem sich anderen Menschen nahe fühlen.

**Ratings for each virtue**

Mir ist diese Tugend wichtig.

Ich zeige diese Tugend wann immer möglich.

Ich habe das Bedürfnis, im Einklang mit dieser Tugend zu handeln.

Es gibt mir ein gutes Gefühl, nach dieser Tugend zu handeln.

Ich finde es richtig, diese Tugend zu zeigen.

Ich fühle mich dieser Tugend stark verpflichtet.

Es ist für mich erfüllend, wenn ich mich im Einklang mit dieser Tugend verhalte.

**Response option for each rating**

«gar nicht» bis «vollkommen» (10 Abstufungen)

**Sample 1: English (tentative translations)**

**Inventory of Core Virtues (ICV-7)**

The present questionnaire deals with the topic of virtue. Virtue is understood to be an outstanding characteristic or exemplary attitude. In the broadest sense, any ability to perform actions considered valuable can be called virtue. In ethics, the term refers to a trait considered important and desirable that enables a person to achieve what is morally good. In the following, six virtues will be presented to you and you will be asked various questions about them. Please note that there are no right or wrong answers – your personal opinion is important to us.

**Virtue descriptions**

Wisdom: Wisdom encompasses a distinct breadth and depth of knowledge about living conditions, human affairs and judgement in the application/implementation of this knowledge. It is not intelligence in the sense of an IQ, but rather practical intelligence that leads to balanced good solutions between self-interest, third party interests and context.

Courage: Courage can be shown in various ways. Physical bravery means overcoming fears of physical injury or death in order to save the lives of others or oneself. Morally brave is a person who maintains ethical integrity and authenticity (fidelity to oneself), even at the risk of losing friends, work, privacy or prestige. Psychological courage is needed to confront a debilitating illness, destructive habits or situations.

Justice: The virtue Justice refers to what we consider as fair. This perception varies depending on cultural background and personal ideas. Justice is often associated with the idea that everyone gets what he or she deserves, that everyone has equal opportunities, and that demands are adapted to need.

Transcendence: The literal meaning comes from the Latin transcendere, which means to exceed. It is what goes beyond human knowledge and understanding. In a broad sense it is the connection to something higher. It is the belief that there is a sense or purpose greater than man himself. In the classical sense, transcendence includes concepts such as religiosity and spirituality. Transcendence can also be triggered by something or someone that makes human concerns and self seem insignificant, and inspires awe, hope and gratitude. Transcendence can also be triggered by something or someone that makes human concerns and self seem insignificant, and inspires to awe, hope and gratitude

Temperance: The virtue of temperance means that one can control excesses. This does not only mean control over desires such as eating, drinking, smoking and sex, but moderation includes any form of successful self-control. Moderation is the overarching ability to control one's own feelings, intentions and behavior.

Humanity: Humanity refers to an interpersonal strength, which shows itself in caring for and helping others. Humanity manifests itself in friendliness, generosity, goodwill and compassion for others. People who show a high degree of this virtue are aware of the feelings and motives of others and themselves and know what to do in different social situations. Humanity also manifests itself in maintaining close relationships and feeling close to others.

**Ratings for each virtue**

This virtue is important to me.

I show this virtue whenever possible.

I feel the need to act in harmony with this virtue.

It gives me a good feeling to act according to this virtue.

I think it is right to show this virtue.

I feel strongly committed to this virtue.

It is fulfilling for me when I act in accordance with this virtue.

**Response option for each rating**

"not at all" to " completely" (10-point scale)

**Sample 2: German (original)**

**Inventory of Core Virtues (ICV-6)**

Der vorliegende Fragebogen befasst sich mit dem Thema Tugend. Im Folgenden werden Ihnen verschiedene Tugenden vorgestellt und Ihnen verschiedene Fragen zu diesen gestellt. Bitte beachten Sie, dass es keine richtigen oder falschen Antworten gibt. Uns ist Ihre persönliche Meinung wichtig.

**Virtue descriptions**

Mut: Mut bezeichnet die Bereitschaft zum Ausüben von Willensleistungen zur Überwindung von inneren und äußeren Barrieren, die der Erreichung von Zielen im Wege stehen, also die Bereitschaft, sich physischen, moralischen und psychischen Widrigkeiten zu stellen.

Gerechtigkeit: Gerechtigkeit bezeichnet das konsequente Befolgen eines unparteiischen Standards der Fairness und Gleichbehandlung und das Eintreten für diesen Standard im Dienste der Gemeinschaft.

Menschlichkeit: Menschlichkeit bezeichnet das über Gerechtigkeit und Fairness hinausgehende prosoziale und altruistische Verhalten im Dienste liebevoller menschlicher Interaktion. Während Gerechtigkeit unparteiisch ist und zuerst der Gemeinschaft dient, ist Menschlichkeit parteiisch und dient zuallererst dem Individuum.

Mäßigung: Mäßigung bezeichnet das Kontrollieren und Beherrschen der eigenen Gefühle, Bedürfnisse und Verhaltensweisen, um Exzessen entgegenzuwirken. Mäßigung ist kurzfristig selbstbeschränkend, bringt jedoch positive Langzeitfolgen für sich selbst und Andere mit sich.

Weisheit und Wissen: Weisheit und Wissen bezeichnet gutes Urteilsvermögen über alle Angelegenheiten des Lebens und das Lernen und den Gebrauch von Wissen, das durch Lebenserfahrungen erworben wird sowie die Fähigkeit zur Reflektion über dieses Wissen und seine Weitergabe.

Transzendenz: Transzendenz bezeichnet den Glauben an und die Verbindung zu einem Zweck, der höher ist als das eigene Selbst. Dieser Glaube und diese Verbindung lassen das eigene Selbst winzig erscheinen, stiften aber gleichzeitig durch das Erheben aus der Unbedeutsamkeit einen höheren Sinn.

**Ratings for each virtue**

- Mir ist diese Tugend wichtig.
- Ich zeige diese Tugend wann immer möglich.
- Ich finde es richtig, diese Tugend zu zeigen.
- Für mich ist diese Tugend irrelevant.
- Ich fühle mich dieser Tugend stark verpflichtet.
- Es ist für mich erfüllend, wenn ich mich im Einklang mit dieser Tugend verhalte.

**Response option for each rating**

«gar nicht» bis «vollkommen» (10 Abstufungen)

**General Virtuousness Rating (GVR)**

Gleiche Instruktion und Antwortskalen wie im ICV-6

Tugendhaftigkeit

Tugendhaftigkeit umfasst, sich an sittliche und gesellschaftliche Normen zu halten und diese zu verinnerlichen. Zudem beinhaltet Tugendhaftigkeit sich anständig, moralisch und vorbildlich zu verhalten. Im weitesten Sinn beinhaltet es auch das Vollbringen als wertvoll betrachteter Leistungen.

- Mir ist es wichtig, tugendhaft zu sein.
- Ich bin wann immer möglich tugendhaft.
- Ich finde es richtig, tugendhaft zu sein.
- Für mich ist Tugendhaftigkeit irrelevant.
- Ich fühle mich der Tugendhaftigkeit stark verpflichtet.
- Es ist für mich erfüllend, wenn ich mich tugendhaft verhalte.

**Sample 2: English (tentative translations)**

**Inventory of Core Virtues (ICV-6)**

The present questionnaire deals with the topic of virtue. In the following, various virtues will be presented to you and you will be asked various questions about them. Please note that there are no right or wrong answers - your personal opinion is important to us.

**Virtue descriptions**

Courage: Courage describes the willingness to exercise willpower to overcome internal and external barriers that stand in the way of achieving goals, i.e. the willingness to face physical, moral and psychological adversities.

Justice: Justice refers to the consistent adherence to an impartial standard of fairness and equal treatment and the advocacy of this standard in the service of the community.

Humanity: Humanity refers to prosocial and altruistic behavior beyond justice and fairness in the service of loving human interaction. While justice is impartial and serves the community first, humanity is biased and serves the individual first.

Temperance: Temperance refers to controlling and mastering one's feelings, needs and behaviors to counteract excesses. Moderation is self-limiting in the short term, but brings positive long-term consequences for oneself and others.

Wisdom and knowledge: Wisdom and knowledge refers to good judgment in all matters of life and the learning and use of knowledge acquired through life experiences and the ability to reflect on and pass on this knowledge.

Transcendence: Transcendence refers to the belief in and connection to a purpose higher than one's own self. This belief and connection make one's own self seem tiny, but at the same time, by rising from insignificance, it creates a higher meaning.

**Ratings for each virtue**

- This virtue is important to me.

- I show this virtue whenever possible.

- I think it is right to show this virtue.

- For me this virtue is irrelevant.

- I feel strongly committed to this virtue.

- It is fulfilling for me to be in harmony with this virtue.

**Response option for each rating**

"not at all" to " completely" (10-point scale)

**General Virtuousness Rating (GVR)**

Same introduction and response options as in the ICV-6

Virtuousness

Virtuousness includes adhering to and internalizing moral and social norms. Furthermore, virtuousness includes behaving in a decent, moral and exemplary manner. In the broadest sense, it also includes the performance of actions considered valuable.

- It is important to me to be virtuous.

- I am virtuous whenever possible.

- I think it is right to be virtuous.

- For me, virtuousness is irrelevant.

- I feel strongly committed to virtuousness.

- It is fulfilling for me to be virtuous.

**Sample 3: German (original)**

**Core Virtue Rating Form (CVRF)**

Lesen Sie bitte die folgenden Beschreibungen zu den Bereichen Mut, Gerechtigkeit, Menschlichkeit, Mäßigung, Weisheit und Wissen, sowie Transzendenz durch. Danach werden Sie gebeten, diese abstrakten Beschreibungen auf sich zu beziehen.

(randomisierte Vorgabe der Tugenden)

Antwortskala: gar nicht (= 1), fast gar nicht (= 2), sehr wenig (= 3), wenig (= 4), teils-teils (= 5), stark (= 6), sehr stark (= 7), fast vollständig (= 8), vollständig (= 9)

Rating pro Tugend: Ihre Aufgabe ist nun, diese abstrakte Aussage auf sich zu beziehen. Wenn Sie sich realistisch und objektiv betrachten: Wie sehr trifft die Beschreibung auf Ihr übliches Verhalten (Handeln, Denken und Fühlen) zu?

**Good-Character Rating (GCR)**

Unter „Charakter“ versteht man oft die Summe bewerteter Persönlichkeitseigenschaften. Im Alltag spricht man dann von einem „guten“ oder „schlechten“ Charakter. Es gibt eine Fülle von Charaktermerkmalen. Schon seit den alten Griechen werden eine Vielzahl von Tugenden und Laster unterschieden. Daher wird wohl jeder Mensch sowohl Charaktermerkmale haben, die er als „gut“ oder „schlecht“ bezeichnet, also diese gerne besitzt und stolz auf sie ist oder eben nicht gerne hat. Bei der folgenden Einschätzung geht es um eine globale Aussage zum eigenen Charakter (also der Summe der als positiv oder negativ bewerteten Eigenschaften).

Zur Einschätzung. Wir gehen davon aus, dass die meisten Menschen charakterlich gut sind. Deswegen wollten wir auf der 9-Punkte Skala mehr Platz für diesen Bereich reservieren. Wir unterteilen auf der folgenden Skala zunächst „schlecht“, „weder noch“ und „gut“ und haben dann für „schlecht“ die Stufen 1-3, für „weder noch“ die Stufe 4 und für „gut“ aber die Stufen 5-9 reserviert.

Wir wissen, dass so eine Einschätzung nur sehr schwierig zu treffen ist und die getätigte Aussage natürlich grob vereinfacht ist und nur sehr global geschehen kann. Aber für die vorliegende Forschungsfrage (wo wir uns auf historische Vorstellungen beziehen) ist eine möglichst realistische und objektive Einschätzung sehr wichtig, auch wenn diese schwer fällt.

Anleitung. Wenn Sie sich mit etwas Distanz und so objektiv wie möglich betrachten: Wie würden Sie Ihren Charakter auf dieser Skala einschätzen?

1 deutlich kein guter Charakter (d.h. sehr lasterhaft)

2 kein guter Charakter (d.h. lasterhaft)

3 eher kein guter Charakter (d.h. eher lasterhaft)

4 weder noch

5 eher guter Charakter (d.h. oft charakterlich gut)

6 guter Charakter (d.h. sehr oft charakterlich gut)

7 sehr guter Charakter (d.h. fast immer charakterlich gut)

8 charakterlich exzellent/herausragend (d.h. immer charakterlich gut)

9 absolut charakterlich exzellent/herausragend (d.h. ausnahmslos charakterlich gut)

**Sample 3: English (tentative translations)**

**Core Virtue Rating Form (CVRF)**

Please read the following descriptions of courage, justice, humanity, moderation, wisdom and knowledge, and transcendence. Afterwards you will be asked to relate these abstract descriptions to yourself.

(randomized specification of the virtues)

Answer scale: not at all (= 1), almost not at all (= 2), very little (= 3), little (= 4), partially (= 5), strongly (= 6), very strongly (= 7), almost completely (= 8), completely (= 9)

Rating per virtue: Your task now is to relate this abstract statement to yourself. If you look at yourself realistically and objectively: To what extent does the description apply to your usual behavior (acting, thinking and feeling)?

**Good-Character Rating (GCR)**

Character" is often understood to be the sum of evaluated personality traits. In everyday life one speaks then of a "good" or "bad" character. There is a wealth of character traits. Since the ancient Greeks, a multitude of virtues and vices have been distinguished. Therefore, every person will probably have character traits that he or she describes as "good" or "bad", i.e. that he or she likes to have and is proud of or does not like. The following assessment is a global statement about one's own character (i.e. the sum of the characteristics evaluated as positive or negative).

To the assessment. We assume that most people are good in character. Therefore, we wanted to reserve more space on the 9-point scale for this area. On the following scale, we first subdivide "bad", "neither yet" and "good" and then reserved levels 1-3 for "bad", level 4 for "neither yet" and levels 5-9 for "good".

We know that such an assessment is very difficult to make and that the statement made is of course grossly simplified and can only be made very globally. But for the present research question (where we refer to historical conceptions) an assessment as realistic and objective as possible is very important, even if it is difficult.

Instructions. If you look at yourself with some distance and as objectively as possible: How would you rate your character on this scale?

1 clearly not a good character (i.e. very vicious)

2 no good character (i.e. vicious)

3 rather no good character (i.e. rather vicious)

4 neither nor

5 rather good character (i.e. displaying good character often)

6 good character (i.e. displaying good character very often)

7 very good character (i.e. displaying good character almost always)

8 of excellent character/outstanding (i.e. always displaying good character)

9 of absolutely excellent character/exemplary (i.e. displaying good character without exception)

**Sample 4:** The same measures as in Sample 3 were employed, with the sole difference that they were worded in the third-person to allow the informant-raters to evaluate the target person.

**Table S1**

***Descriptive statistics of the scales (POMP) in the four samples and in the total sample***

|  | Sample 1  (*N* = 260) | | | Sample 2  (*N* = 378) | | | Sample 3  (*N* = 259) | |  | Sample 4  (*N* = 344) | | Total  (*N* = 981-1241) | |
| --- | --- | --- | --- | --- | --- | --- | --- | --- | --- | --- | --- | --- | --- |
| VIA scales | *M* | *SD* | α | *M* | *SD* | α | *M* | *SD* |  | *M* | *SD* | *M* | *SD* |
| Character strengths |  |  |  |  |  |  |  |  |  |  |  |  |  |
| Creativity | 66.06 | 23.13 | - | 58.47 | 18.15 | .89 | 59.49 | 17.70 | .90 | 71.91 | 22.59 | 64.00 | 21.21 |
| Curiosity | 75.87 | 20.06 | - | 69.19 | 13.67 | .79 | 70.65 | 12.75 | .78 | 76.74 | 20.88 | 72.99 | 17.51 |
| Judgment | 75.00 | 20.07 | - | 71.82 | 12.86 | .81 | 71.11 | 12.14 | .80 | 75.36 | 22.32 | 73.32 | 17.50 |
| Love of learning | 72.02 | 21.09 | - | 65.21 | 16.46 | .84 | 65.69 | 15.10 | .82 | 76.85 | 21.51 | 69.97 | 19.38 |
| Perspective | 69.33 | 19.19 | - | 65.49 | 12.40 | .77 | 65.07 | 12.67 | .80 | 69.69 | 20.93 | 67.37 | 16.82 |
| Bravery | 61.59 | 22.21 | - | 60.26 | 13.76 | .76 | 63.09 | 13.94 | .79 | 66.50 | 23.56 | 62.86 | 18.99 |
| Perseverance | 68.46 | 21.23 | - | 61.28 | 15.82 | .86 | 62.04 | 15.78 | .87 | 75.33 | 22.94 | 66.84 | 20.06 |
| Honesty | 76.92 | 17.61 | - | 69.48 | 11.62 | .74 | 70.77 | 11.11 | .74 | 85.72 | 16.20 | 75.81 | 15.78 |
| Zest | 66.30 | 19.39 | - | 60.96 | 14.89 | .81 | 61.07 | 14.22 | .79 | 68.28 | 22.74 | 64.13 | 18.49 |
| Love | 75.19 | 20.81 | - | 71.22 | 15.12 | .79 | 73.42 | 14.31 | .79 | 80.05 | 21.07 | 74.96 | 18.37 |
| Kindness | 77.88 | 17.85 | - | 69.35 | 13.20 | .76 | 71.49 | 11.50 | .71 | 81.87 | 18.74 | 75.05 | 16.47 |
| Social intelligence | 76.44 | 18.87 | - | 68.98 | 13.13 | .80 | 70.13 | 12.68 | .80 | 80.23 | 18.31 | 73.90 | 16.61 |
| Teamwork | 66.88 | 22.39 | - | 66.20 | 12.90 | .75 | 67.04 | 12.01 | .74 | 70.53 | 22.58 | 67.72 | 18.15 |
| Fairness | 77.16 | 17.28 | - | 72.30 | 13.16 | .79 | 72.08 | 11.94 | .76 | 81.14 | 17.63 | 75.73 | 15.67 |
| Leadership | 65.38 | 22.24 | - | 65.71 | 12.99 | .79 | 65.96 | 11.92 | .75 | 67.01 | 22.98 | 66.05 | 18.18 |
| Forgiveness | 69.38 | 19.99 | - | 60.56 | 15.48 | .83 | 60.71 | 13.98 | .80 | 72.06 | 20.58 | 65.62 | 18.48 |
| Humility | 62.93 | 21.28 | - | 55.60 | 13.53 | .76 | 55.34 | 14.37 | .79 | 69.88 | 24.46 | 61.04 | 19.92 |
| Prudence | 62.16 | 24.91 | - | 61.59 | 14.73 | .80 | 60.32 | 14.64 | .81 | 66.35 | 24.63 | 62.76 | 20.33 |
| Self-regulation | 58.56 | 22.91 | - | 54.19 | 15.04 | .76 | 54.92 | 14.99 | .75 | 60.25 | 25.87 | 56.94 | 20.42 |
| Beauty | 69.86 | 20.97 | - | 60.56 | 15.11 | .77 | 60.62 | 15.51 | .79 | 70.86 | 20.72 | 65.37 | 18.83 |
| Gratitude | 70.00 | 20.14 | - | 68.10 | 14.16 | .81 | 68.92 | 13.73 | .82 | 73.40 | 19.91 | 70.14 | 17.30 |
| Hope | 69.38 | 21.02 | - | 62.12 | 15.64 | .81 | 63.45 | 14.91 | .80 | 69.77 | 20.58 | 66.04 | 18.50 |
| Humor | 73.94 | 19.22 | - | 64.82 | 16.19 | .87 | 65.54 | 15.47 | .87 | 74.82 | 20.94 | 69.65 | 18.71 |
| Spirituality | 41.15 | 30.99 | - | 38.43 | 21.84 | .89 | 39.43 | 23.08 | .91 | 39.17 | 31.34 | 39.41 | 27.00 |
| Virtues |  |  |  |  |  |  |  |  |  |  |  |  |  |
| Wisdom/knowledge | 72.95 | 16.58 | .93 | 81.01 | 15.21 | .87 | 65.15 | 16.12 | - | 66.86 | 16.53 | 72.09 | 17.30 |
| Courage | 65.48 | 18.73 | .94 | 69.86 | 17.60 | .87 | 59.56 | 17.72 | - | 62.21 | 16.97 | 64.67 | 18.11 |
| Humanity | 83.27 | 14.63 | .93 | 84.65 | 16.35 | .92 | 67.95 | 17.15 | - | 71.00 | 17.66 | 77.09 | 18.07 |
| Justice | 82.25 | 14.82 | .94 | 85.74 | 15.03 | .90 | 67.57 | 16.33 | - | 71.48 | 16.80 | 77.26 | 17.44 |
| Temperance | 59.75 | 19.48 | .94 | 63.87 | 19.96 | .90 | 55.60 | 18.08 | - | 58.10 | 19.10 | 59.68 | 19.47 |
| Transcendence | 41.28 | 28.83 | .98 | 44.39 | 27.29 | .95 | 39.24 | 27.01 | - | 40.44 | 25.66 | 41.57 | 27.17 |
| Good character | - | - | - | 60.10 | 14.53 | .93 | 66.89 | 13.72 | - | 76.74 | 11.75 | 67.73 | 15.18 |

*Note.* Beauty = Appreciation of beauty and excellence.

**Table S2**

***Inter-Correlations of the core virtues***

| Core virtues | (1) | (2) | (3) | (4) | (5) |
| --- | --- | --- | --- | --- | --- |
| Sample 1 (*N* = 260) |  |  |  |  |  |
| (2) Courage | .30 |  |  |  |  |
| (3) Humanity | .37 | .38 |  |  |  |
| (4) Justice | .34 | .33 | .58 |  |  |
| (5) Temperance | .30 | .22 | .22 | .24 |  |
| (6) Transcendence | .12 | .13 | .25 | .09 | .18 |
| Sample 2 (*N* = 378) |  |  |  |  |  |
| (2) Courage | .41 |  |  |  |  |
| (3) Humanity | .30 | .23 |  |  |  |
| (4) Justice | .38 | .27 | .52 |  |  |
| (5) Temperance | .30 | .26 | .19 | .27 |  |
| (6) Transcendence | .16 | .17 | .12 | .04 | .17 |
| Sample 3 (*N* = 259) |  |  |  |  |  |
| (2) Courage | .26 |  |  |  |  |
| (3) Humanity | -.03 | .22 |  |  |  |
| (4) Justice | .16 | .24 | .36 |  |  |
| (5) Temperance | .33 | .13 | -.01 | .18 |  |
| (6) Transcendence | .04 | .14 | .10 | .10 | .05 |
| Sample 4 (*N* = 344) |  |  |  |  |  |
| (2) Courage | .25 |  |  |  |  |
| (3) Humanity | .22 | .16 |  |  |  |
| (4) Justice | .28 | .18 | .40 |  |  |
| (5) Temperance | .28 | .22 | .16 | .20 |  |
| (6) Transcendence | .18 | .12 | .21 | .23 | .22 |
| Total sample (*N* = 1,241) |  |  |  |  |  |
| (2) Courage | .36 |  |  |  |  |
| (3) Humanity | .32 | .29 |  |  |  |
| (4) Justice | .40 | .31 | .55 |  |  |
| (5) Temperance | .33 | .24 | .19 | .26 |  |
| (6) Transcendence | .15 | .15 | .18 | .13 | .17 |

*Notes.* (1) = wisdom and knowledge.

**Table S3**

***Zero-Order Correlations between the character strengths and virtue ratings across the four samples***

| CS | Wisdom | Courage | Humanity | Justice | Temperance | Transcendence | *Mdn* |
| --- | --- | --- | --- | --- | --- | --- | --- |
| Creativity | .13 | .15 | .00 | .02 | .02 | .07 | .02 |
| Curiosity | .15 | .17 | .04 | .12 | .09 | .07 | .09 |
| Judgment | .28 | .08 | .06 | .15 | .17 | .00 | .08 |
| Learning | .18 | .13 | -.03 | .07 | .12 | .06 | .07 |
| Perspective | .32 | .11 | .11 | .14 | .17 | .10 | .11 |
| Bravery | .10 | .38 | .03 | .07 | .10 | .08 | .08 |
| Perseverance | .08 | .15 | .02 | .06 | .23 | .04 | .06 |
| Honesty | .06 | .12 | .10 | .17 | .13 | .08 | .10 |
| Zest | .09 | .22 | .08 | .08 | .11 | .07 | .08 |
| Love | .04 | .06 | .24 | .12 | .03 | .11 | .06 |
| Kindness | .01 | .04 | .30 | .19 | .08 | .10 | .08 |
| Social Intelligence | .10 | .09 | .22 | .13 | .14 | .10 | .10 |
| Teamwork | -.01 | .06 | .18 | .15 | .12 | .05 | .06 |
| Fairness | .07 | .11 | .24 | .35 | .16 | .09 | .11 |
| Leadership | .14 | .18 | .11 | .13 | .17 | .07 | .14 |
| Forgiveness | .02 | .05 | .16 | .13 | .18 | .15 | .13 |
| Humility | -.02 | -.04 | .13 | .17 | .21 | .13 | .13 |
| Prudence | .14 | -.04 | .10 | .16 | .27 | .07 | .10 |
| Self-regulation | .11 | .19 | .01 | .08 | .38 | .10 | .10 |
| Beauty | .10 | .12 | .12 | .09 | .11 | .24 | .11 |
| Gratitude | .08 | .13 | .23 | .19 | .14 | .26 | .14 |
| Hope | .06 | .18 | .06 | .06 | .11 | .09 | .06 |
| Humor | .05 | .10 | .18 | .10 | .03 | -.01 | .10 |
| Spirituality | .03 | .11 | .07 | .02 | .07 | .61 | .07 |

*Notes. N* = 1,241. CS = character strengths, Learning = love of learning, Beauty = Appreciation of beauty and excellence.

***Table S4***

***Individual correlations of the 24 character strengths with the 6 core virtues (controlling for age and gender)***

|  | Sample 1 (*df* = 254) | | | | | | | Sample 2 (*df* = 374) | | | | | | | Sample 3 (*df* = 255) | | | | | | | Sample 4 (*df* = 340) | | | | | | |
| --- | --- | --- | --- | --- | --- | --- | --- | --- | --- | --- | --- | --- | --- | --- | --- | --- | --- | --- | --- | --- | --- | --- | --- | --- | --- | --- | --- | --- |
| CS | W | C | H | J | Te | Tr | *Mdn* | W | C | H | J | Te | Tr | *Mdn* | W | C | H | J | Te | Tr | *Mdn* | W | C | H | J | Te | Tr | *Mdn* |
| Creativity | .09 | .03 | .04 | .11 | -.08 | .03 | .03 | .13 | .15 | .01 | -.01 | .04 | .05 | .04 | .23 | .33 | .00 | .01 | .04 | .21 | .04 | .31 | .22 | .10 | .16 | .12 | .03 | .12 |
| Curiosity | .16 | .04 | .12 | .19 | .00 | -.01 | .04 | .14 | .18 | .05 | .11 | .10 | .06 | .10 | .21 | .42 | .08 | .13 | .10 | .19 | .13 | .30 | .21 | .07 | .20 | .18 | .05 | .18 |
| Judgment | .17 | .01 | .19 | .15 | .07 | -.05 | .07 | .25 | .03 | .00 | .13 | .15 | -.01 | .03 | .48 | .30 | .09 | .19 | .19 | .01 | .19 | .40 | .09 | .07 | .25 | .26 | .06 | .09 |
| Learning | .14 | .11 | .09 | .15 | .14 | -.05 | .11 | .24 | .14 | -.02 | .02 | .00 | .07 | .02 | .30 | .25 | -.11 | .09 | .11 | .17 | .11 | .34 | .13 | .05 | .21 | .25 | .04 | .13 |
| Perspective | .27 | .04 | .12 | .12 | .17 | .07 | .12 | .25 | .05 | .11 | .08 | .13 | .06 | .08 | .50 | .36 | .12 | .22 | .17 | .13 | .17 | .47 | .13 | .20 | .24 | .24 | .15 | .20 |
| Bravery | .04 | .34 | .07 | .17 | .11 | .02 | .07 | .10 | .23 | .06 | .04 | .02 | .19 | .06 | .23 | .56 | .08 | .25 | .08 | .14 | .14 | .22 | .55 | .12 | .11 | .18 | .01 | .12 |
| Perseverance | -.02 | .08 | -.01 | .07 | .19 | -.07 | -.01 | .15 | .21 | .07 | .13 | .19 | .05 | .13 | .22 | .35 | .09 | .21 | .25 | .05 | .21 | .24 | .20 | .14 | .12 | .35 | .08 | .14 |
| Honesty | .12 | .19 | .12 | .28 | .21 | .10 | .12 | .14 | .11 | .27 | .26 | .16 | .10 | .16 | .20 | .33 | .10 | .37 | .21 | .08 | .20 | .23 | .14 | .30 | .33 | .13 | .09 | .23 |
| Zest | .03 | .09 | .05 | .08 | .08 | -.04 | .05 | .11 | .21 | .04 | .04 | .05 | .07 | .05 | .12 | .31 | .15 | .11 | .14 | .11 | .12 | .21 | .34 | .16 | .18 | .20 | .09 | .18 |
| Love | .17 | -.02 | .30 | .28 | .05 | .09 | .09 | .03 | .18 | .27 | .18 | .07 | .12 | .12 | .05 | .19 | .25 | .05 | -.02 | .06 | .05 | .13 | .03 | .37 | .17 | .07 | .14 | .13 |
| Kindness | .09 | .02 | .30 | .28 | .11 | .05 | .09 | .08 | .14 | .44 | .26 | .14 | .09 | .14 | -.03 | .14 | .33 | .30 | .06 | .14 | .14 | .15 | .05 | .47 | .28 | .12 | .16 | .15 |
| Social Int. | .05 | .00 | .32 | .24 | .08 | .05 | .05 | .16 | .16 | .26 | .11 | .12 | .11 | .12 | .27 | .38 | .31 | .25 | .09 | .12 | .25 | .23 | .05 | .29 | .20 | .29 | .12 | .20 |
| Teamwork | -.14 | -.05 | -.04 | .02 | .02 | -.03 | -.04 | .07 | .19 | .36 | .31 | .25 | .09 | .19 | .00 | .16 | .29 | .22 | .16 | .03 | .16 | .11 | .08 | .32 | .24 | .15 | .13 | .13 |
| Fairness | .10 | .21 | .25 | .39 | .12 | .02 | .12 | .10 | .15 | .45 | .42 | .23 | .17 | .17 | .09 | .15 | .13 | .41 | .17 | .04 | .13 | .18 | .06 | .32 | .51 | .17 | .13 | .17 |
| Leadership | .02 | .05 | -.07 | -.05 | .09 | -.02 | .02 | .20 | .19 | .34 | .34 | .23 | .18 | .20 | .16 | .37 | .30 | .31 | .15 | .12 | .16 | .25 | .25 | .10 | .15 | .21 | .02 | .21 |
| Forgiveness | .04 | .07 | .22 | .16 | .25 | .12 | .12 | .06 | .12 | .31 | .17 | .16 | .22 | .17 | -.03 | .15 | .14 | .15 | .15 | .10 | .14 | .17 | -.01 | .15 | .27 | .22 | .14 | .15 |
| Humility | .01 | -.02 | .21 | .18 | .28 | .16 | .16 | -.08 | -.04 | .18 | .17 | .14 | .05 | .05 | -.02 | -.09 | .12 | .25 | .31 | .04 | .04 | .17 | .05 | .25 | .37 | .26 | .19 | .19 |
| Prudence | .06 | .04 | .16 | .19 | .28 | .09 | .09 | .16 | -.03 | .12 | .22 | .18 | .06 | .12 | .29 | .02 | .12 | .26 | .28 | .04 | .12 | .20 | -.14 | .13 | .17 | .37 | .07 | .13 |
| Regulation | .04 | .17 | .01 | .05 | .38 | .11 | .05 | .11 | .18 | .04 | .10 | .21 | .08 | .10 | .22 | .27 | .05 | .16 | .39 | .06 | .16 | .20 | .23 | .08 | .17 | .57 | .10 | .17 |
| Beauty | .12 | .09 | .18 | .11 | .12 | .26 | .12 | .10 | .14 | .17 | .07 | .06 | .22 | .10 | .07 | .17 | .14 | .12 | .00 | .33 | .12 | .29 | .18 | .14 | .20 | .23 | .18 | .20 |
| Gratitude | .05 | .08 | .24 | .24 | .16 | .15 | .16 | .09 | .19 | .27 | .11 | .13 | .26 | .13 | -.01 | .20 | .25 | .13 | .06 | .32 | .13 | .25 | .17 | .31 | .39 | .22 | .31 | .25 |
| Hope | .00 | .10 | .12 | .14 | .08 | .00 | .10 | .09 | .21 | .08 | .07 | .05 | .15 | .08 | .10 | .25 | .11 | -.07 | .16 | .07 | .11 | .19 | .24 | .09 | .16 | .17 | .09 | .17 |
| Humor | .09 | .04 | .20 | .06 | -.04 | .01 | .06 | .12 | .14 | .25 | .15 | .13 | -.02 | .14 | .00 | .23 | .27 | .14 | .00 | .05 | .14 | .15 | .15 | .20 | .19 | .08 | .06 | .15 |
| Spirituality | -.12 | .07 | .11 | -.03 | .03 | .60 | .03 | .09 | .15 | .05 | -.05 | .05 | .56 | .05 | -.02 | .13 | .10 | .07 | .06 | .76 | .07 | .12 | .10 | .07 | .07 | .09 | .56 | .09 |

*Note.* *Mdn* = median correlation across the 5 non-assigned virtues. CS = Character strengths, Learning = love of learning, Social Int. = social intelligence, Regulation = self-regulation, Beauty = Appreciation of beauty and excellence.

**Table S5**

***Zero-Order Correlations between the Character Strengths and the “Good Character” Ratings***

| CS | Total sample  (*N* = 978) | Sample 2  (*n* = 378) | Sample 3  (n = 259) | Sample 4  (*n* = 344) |
| --- | --- | --- | --- | --- |
| Creativity | .22 | -.11 | .16 | .29 |
| Curiosity | .23 | .05 | .32 | .20 |
| Judgment | .16 | .05 | .20 | .19 |
| Learning | .20 | -.04 | .16 | .19 |
| Perspective | .23 | .06 | .35 | .25 |
| Bravery | .15 | -.03 | .21 | .15 |
| Perseverance | .37 | .26 | .23 | .32 |
| Honesty | .42 | .22 | .28 | .30 |
| Zest | .27 | .08 | .33 | .31 |
| Love | .29 | .09 | .39 | .25 |
| Kindness | .38 | .25 | .23 | .33 |
| Social Int. | .34 | .07 | .36 | .34 |
| Teamwork | .27 | .33 | .26 | .21 |
| Fairness | .35 | .19 | .22 | .39 |
| Leadership | .20 | .21 | .30 | .21 |
| Forgiveness | .26 | .11 | .21 | .15 |
| Humility | .34 | .18 | .16 | .32 |
| Prudence | .25 | .29 | .24 | .19 |
| Regulation | .22 | .15 | .26 | .20 |
| Beauty | .25 | .06 | .23 | .19 |
| Gratitude | .29 | .17 | .30 | .34 |
| Hope | .26 | .08 | .35 | .24 |
| Humor | .24 | .06 | .24 | .20 |
| Spirituality | .09 | .08 | .18 | .06 |

*Notes.* CS = character strengths, Learning = love of learning, Social Int. = social intelligence, Regulation = self-regulation, Beauty = Appreciation of beauty and excellence.
